# Autonomous Microrobotic Manipulation Using Visual Servo Control

**DOI:** 10.3390/mi11020132

**Published:** 2020-01-24

**Authors:** Matthew Feemster, Jenelle A. Piepmeier, Harrison Biggs, Steven Yee, Hatem ElBidweihy, Samara L. Firebaugh

**Affiliations:** 1 Weapons, Robotics, and Control Engineering Department, United States Naval Academy, Annapolis, MD 21402, USA; feemster@usna.edu (M.F.); piepmeie@usna.edu (J.A.P.); 2 Electrical and Computer Engineering Department, United States Naval Academy, Annapolis, MD 21402, USA; hbiggsstem2019@gmail.com (H.B.); syee@usna.edu (S.Y.); elbidwei@usna.edu (H.E.)

**Keywords:** autonomous robots, micromanipulators, mobile robots, robot control, microassembly

## Abstract

This describes the application of a visual servo control method to the microrobotic manipulation of polymer beads on a two-dimensional fluid interface. A microrobot, actuated through magnetic fields, is utilized to manipulate a non-magnetic polymer bead into a desired position. The controller utilizes multiple modes of robot actuation to address the different stages of the task. A filtering strategy employed in separation mode allows the robot to spiral from the manipuland in a fashion that promotes the manipulation positioning objective. Experiments demonstrate that our multiphase controller can be used to direct a microrobot to position a manipuland to within an average positional error of approximately 8 pixels (64 µm) over numerous trials.

## 1. Introduction

Microrobotic systems offer the capability to revolutionize healthcare and bioengineering [1,2] and also are an enabling technology for additive micromanufacturing [3]. A number of groups have demonstrated the use of magnetically actuated microrobots to manipulate microscale objects in two-dimensions, using pushing [4,5], capillary gripping [6], or trapping through a locally generated flow field [7,8]. This work is concerned with the challenge of autonomous control for an electromagnetic microrobot manipulator based on pushing. Surface forces, such as friction or viscous drag, that counteract volumetric forces such as magnetic pull or inertia are inherently difficult to quantify at the microscale. Furthermore, as a microrobot interacts with a manipuland, the robot and manipuland now form a new, interconnected system with similarly uncertain dynamics. We seek to promote accurate manipulation of a manipuland by a microrobot in spite of uncertain or inaccurate models at the microscale.

A number of varying control strategies have been applied to magnetic microrobot systems [9,10,11,12]. One recent methodology is online estimation of controller parameters using iterative methods such as Broyden’s method or recursive nonlinear least squares estimation. Such uncalibrated adaptive methods have been successfully implemented in macro-scale manipulators and mobile robots for a variety of applications with more complex nonlinear system models and higher degrees of freedoms (DOF) [13,14,15]. Our group has previously demonstrated an adaptive microrobot control scheme in two- [16] and three- [17] dimensions that uses recursive least squares (RLS) estimation to adaptively derive the relationship of the robot velocity to the control signal.

Banerjee and Gupta provide a review of research in automated planning and control for micromanipulation in [18]. A variety of groups [19,20,21,22,23,24,25,26] have demonstrated micromanipulation of one or more manipulands in fluidic environments. Table 1 compares a number of these works, looking at the types and amount of actuation, path planning, feedback control, release strategy, and positioning accuracy. It is worth noting that none of these works demonstrate a complete sequence of manipulation, closed-loop positioning, and release of a manipuland or microobject. 

We use a behavior-based planner [27] similar to that described in [28] and [29], in which we have decomposed the manipulation task into multiple stages. Similar to the microassembly work in [30], the phases are part of a heuristic control strategy defined by robot positions relative to intermediate goal and failure regions. By varying the commanded robot position for each phase, we are able to autonomously direct the microrobot to push the manipuland into position. Many groups have demonstrated this approach [20,22,24,25,26]. The separation of the robot from the manipuland once the manipuland has been brought into position (termed exit stage) can be problematic in micromanipulation systems in which van der Waals forces and local flow fields can cause the manipuland to retract with the microrobot [20,26]. Few groups have developed a systematic approach to address the exit stage. In [5], Pawashe et al. utilized an iterative learning control approach to estimate a compensation distance so that when the robot retracted, the microsphere remained close to the desired location. Though validation is presented in [5], the iterative learning algorithm could be disrupted if an unmodeled/external disturbance is experienced during the learning phase of the controller or if robot motion is not consistent/periodic. 

Therefore, we propose a feedback-driven escape vector approach to extract the robot from the manipuland in a manner to reduce displacement of the manipuland from its desired position. Specifically, the robot is commanded to move to a select radius away from manipuland along a path that promotes movement of the manipuland toward the goal position. The complete process is evaluated on an experimental testbed. Thus, from arbitrary positions in the fluidic workspace, the system autonomously controls the microrobot towards an effective position to move the manipuland using either contact or noncontact pushing or pulling towards a designated target. The system is agnostic to the type of manipulation that is occurring (contact pushing or noncontact pushing or pulling) as it monitors the relative positions of the microrobot and the manipuland. The completely closed loop system positions the manipuland and moves away from the manipuland while maintaining a manipuland positioning accuracy that averages 33% of the manipuland diameter (demonstrated on two different manipuland sizes). 

## 2. Experimental Setup

**Figure 1:**
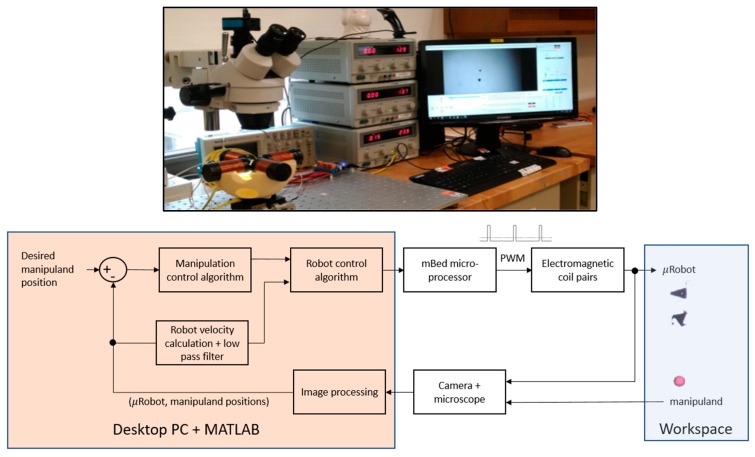
Photograph (**top**) of microrobot test station and block diagram (**bottom**) of the control system.

The electromagnetic microrobot system, illustrated in Figure 1, utilizes a ferromagnetic microrobot suspended at a fluid interface. The microrobot is actuated through attractive magnetic field gradients generated by two electromagnet pairs. For the subsequent manipulation work, two micro-robots are utilized to manipulate polymer microspheres of various size. Initially, a triangular (wedge), nickel structure fabricated through the MEMSCAP® MetalMUMPS process approximately 250 µm in length and 20 µm thick was employed to manipulate a 200-µm-diameter red polymer microsphere (ChromoSphere^TM^-T – see Figure 1). The triangle, which had the greatest nickel mass of the MetalMUMPS designs tested, was used for the initial set of studies because it was the most responsive to magnetic fields. It also has a small contact surface with the manipuland, easing the process of separating the robot from the manipuland. In order to demonstrate the robustness of the approach, a star shaped robot of similar in size (see Figure 1) was substituted for the wedge microrobot in a subsequent experimental trial. The robot and manipuland positions are measured at a frequency of approximately 20 Hz and they are calculated by morphology and color on images captured via a microscope-focused camera to delineate the robot/manipuland within the MATLAB software environment. Note that the described experiments are conducted in the image workspace (i.e., the robot/manipuland’s position are expressed in pixels as opposed to microns). At the magnification level used in these experiments, a pixel corresponds to approximately 8 μm, and the total field of view was 6.0 × 3.8 mm. In addition, the robot’s velocity in the workspace is calculated by employing a backwards difference approach to the robot’s position and passing the result through a low pass filter set to a cutoff frequency of 5 Hz.

The two primary components of the control system are: (1) the Manipulation Control Algorithm and (2) the Robot Control Algorithm. As an overview, the Manipulation Control Algorithm accepts manipulation error as its input and then calculates a two-dimensional, desired robot position that promotes the manipulation objective (this algorithm is presented in detail in Section 3). The Robot Control Algorithm is responsible for calculating the appropriate pulse width modulated (PWM) signals to apply to each of the four electromagnets such that the robot’s velocity ($u_{x},u_{y}$) vector is tracking towards the commanded position.

## 3. Manipulation Control Algorithm

**Figure 2:**
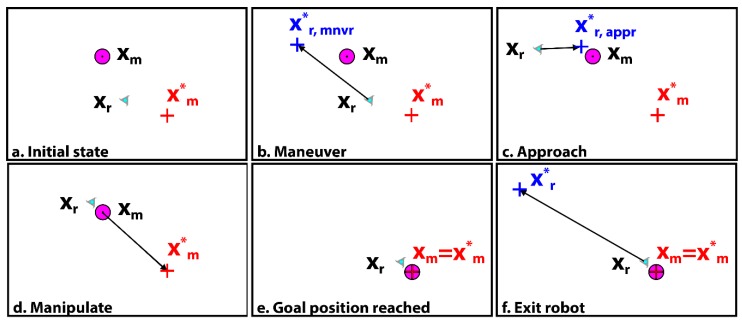
Illustration of manipulation modes. From the initial state (**a**), the system proceeds to the maneuver mode (**b**) where the robot is directed around the back of the manipuland to a position, $x_{r,mnvr}^{*}$. The approach mode (**c**) is utilized when the robot is far from the manipuland but not between the manipuland and the target, and the robot is directed to come into close proximity with the manipuland from behind at position $x_{r,appr}^{*}$. In the manipulate mode, (**d**) the robot drives the manipuland towards the target, $x_{m}^{*}$. Once the manipuland target position is reached (**e**), the exit mode (**f**) is used to drive the robot away from the manipuland.

Our non-sequential phased approach to the micromanipulation task is illustrated in Figure 2, in which the robot is represented by the triangle with a centroid at position $x_{r}$, the manipuland is the red circle with centroid position $x_{m},$ and the target location is the cross with position $x_{m}^{*}$. Five phases or modes are defined. The ‘maneuver’ mode is used to bring the robot around to the back of the manipuland, and sets the goal position at the intermediate point, $x_{r,mnvr}^{*}$, from which the microrobot can subsequently approach the manipuland without driving the manipuland further away from the manipuland target location. The ‘approach’ mode is used to bring the robot into a position near to the manipuland and directly opposite the manipuland target location, at a position labeled $x_{r,appr}^{*}$. 

Once the robot is brought within a specified tolerance of $x_{r,appr}^{*}$ (denoted by $d_{tol}$ in the following table), the controller then switches to the ‘manipulate’ mode, in which the algorithm commands the microrobot to transition along the manipuland error vector towards $x_{m}^{*}$ (effectively, the robot is attempting to move towards $x_{m}^{*}$ through the manipuland). With the calculations of the manipuland error vector updated each control cycle, the robot is able to push along a path that reduces the manipuland error. 

Once the manipuland is brought to within a specified tolerance of the target position $x_{m}^{*}$, the controller switches to an ‘exit’ mode wherein the robot is directed away from the manipuland. If the microrobot simply attempts to move away, the manipuland often is ‘pulled’ along with moving fluid. In order to prevent such displacement of the manipuland away from $x_{m}^{*}$, the proposed separation mode attempts to move the robot to a specified radius out (large enough that robot movement minimally influences the manipuland) from the target location $x_{m}^{*}$ in a manner (filtered) that promotes a reduction of the manipuland error. If the manipuland is pulled away from $x_{m}^{*}$ then the robot’s desired position is set to the opposite side of the manipuland such that the manipuland is driven back towards $x_{m}^{*}$. Eventually, through this closed-loop process, the robot spirals to a distance from $x_{m}^{*}$ where its movement does not significantly influence the manipuland and can be driven to its final ‘parking point’ in a select corner of the image workspace.

Table 2 summarizes the commanded robot positions that are used by each controller mode, and Table 3 provides the underlying equations calculate the critical positions.

**Figure 3:**
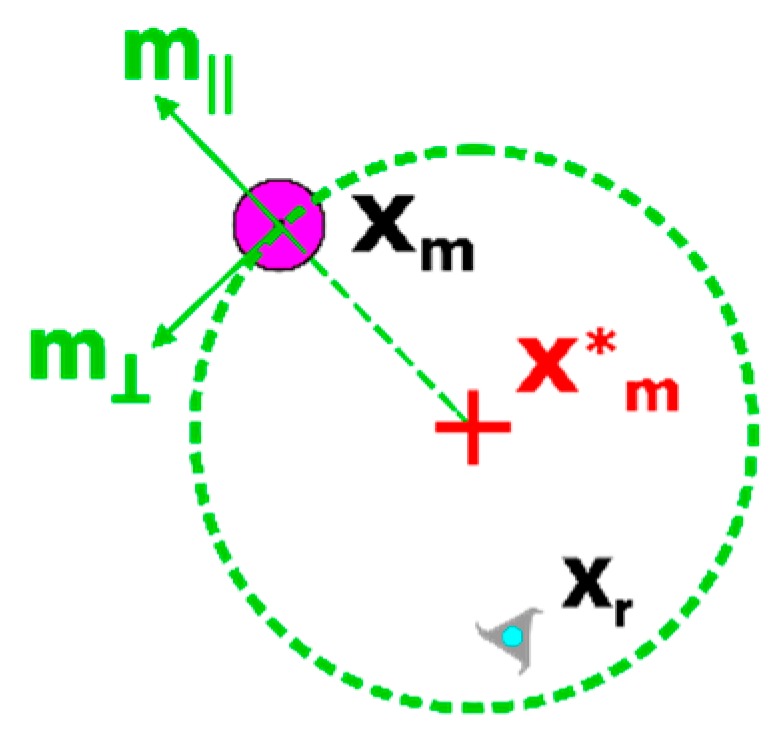
Detail of vector quantities used for critical point determination. The critical points are referenced to the manipuland position by the use of the $m_{⊥}$ and $m_{∥}$ vectors, which are unit vectors representing the radial and tangential components, respectively, of the manipuland position $x_{m}$ relative to the desired manipuland position $x_{m}^{*}$.

The equations for $x_{r,appr}^{*}$ and $x_{r,mnvr}^{*}$ make use of the unit vectors $m_{∥}$ and $m_{⊥}$ (illustrated in Figure 3) which capture the radial and tangential directions with respect to a circle circumscribing the manipuland target position $x_{m}^{*}$, with a radius equal to $\left|x_{m}-x_{m}^{*}\right|$. Tunable parameters $d_{∥}$, $d_{⊥}$, $d_{app}$, $d_{m}$, and $d_{r}$ are selected by the operator before executing the controller.

During manipulation mode, if the gradient of the normalized manipuland error $\left|x_{m}-x_{m}^{*}\right|$ is increasing for more than 0.5s (i.e., the manipuland to moving away from the target position), the mode is switched back to maneuver mode. Since such obstacle avoidance approaches as [31] are not currently implemented, the robot is safely commanded back to a maneuver point to prevent interfering with the manipuland upon reset.

**Figure 4:**
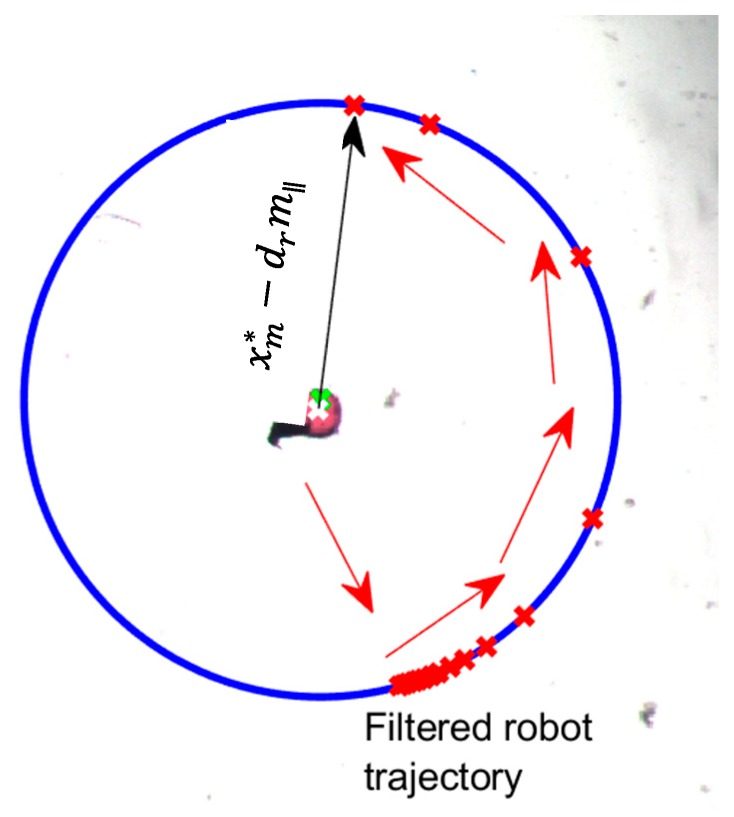
Illustration of filtered path for exit/separation of robot from manipuland.

Based directly on Equation (6), the robot exit/separation mode attempts to move to a desired radius $d_{r}$ out from $x_{m}^{*}$ along $m_{∥}$. In essence, this represents a direct oscillatory (push/pull) robot motion along $m_{∥}$ in an attempt to eventually separate the robot from the manipuland. Though moderately effective, this approach produced somewhat aggressive robot motion while also having the potential for lengthy oscillations if the manipuland moves back and forth across $x_{m}^{*}$. To alleviate these issues, the following low pass filter was utilized to smooth the robot’s commanded position

(1) $$G\left(s\right)=\frac{x_{r,exit}^{*}}{x_{m}^{*}-d_{r}m_{∥}}=\frac{\alpha }{\left(s+\alpha \right)}$$

where α represents a user selected time constant. By employing the filter of Equation (7), the robot now attempts to track an arcing (spiraling) motion along $d_{r}$ arriving at $x_{m}^{*}-d_{r}m_{∥}$ at a time specified by the value of α (assuming the manipuland position remains constant). An example of the approach can be observed in Figure 4 for a value of α=1 (approximately 4s settling time).

## 4. Robot Control Algorithm

The primary objective of the robot control algorithm is to achieve set point regulation of the robot to the desired position provided by the Manipulation Control Algorithm. To this end, many control algorithms have been investigated [9,10,11,12] for microrobots to provide positioning capabilities. In an effort to highlight the manipulation control strategy while simultaneously attempting to account for unmodeled dynamics of the microrobot system, the following proportional integral (PI) robot velocity controller was employed to drive the robot towards the commanded position

(2) $$e_{u}=k_{p}\left(x_{r}^{*}-x_{r}\right)-u_{r}$$

(3) $$dc={\left[dc_{x}\;dc_{y}\;\right]}^{T}=k_{u}e_{u}+k_{i}\int_{0}^{t}e_{u}dt$$

where $e_{u}$ is the robot velocity tracking error, $u_{r}$ represents the robot’s measured velocity signal, dc is the signed duty cycle input, and $k_{p}$, $k_{u}$, and $k_{i}$ denote positive, constant, control gains. Since the system of Figure 1 is equipped with an opposing pair arrangement of electro magnets, the following allocation scheme is utilized in order to properly energize each coil

(4) $$dc_{N}=\left(dc_{y}/2\right)\cdot \left(1+sign\left(dc_{y}\right)\right)$$

(5) $$dc_{S}=\left(dc_{y}/2\right)\cdot \left(1-sign\left(dc_{y}\right)\right)$$

(6) $$dc_{S}=\left(dc_{y}/2\right)\cdot \left(1-sign\left(dc_{y}\right)\right)$$

(7) $$dc_{W}=\left(dc_{x}/2\right)\cdot \left(1-sign\left(dc_{x}\right)\right)$$

where $dc_{N},\;dc_{S},\;dc_{E},\;and\;dc_{W}$ represents the individual duty cycles applied to the north, south, east, and west electromagnets, respectively. 

## 5. Results and Discussion

For the experimental trials, the manipuland’s desired final position was selected as (372,240) pixels (approximately the center of image workspace). The manipuland and the microrobot were actuated to random initial positions within the workspace prior to initiating the control sequence. Table 4 summarizes the parameters and control gains utilized for the experiments. 

**Figure 5:**
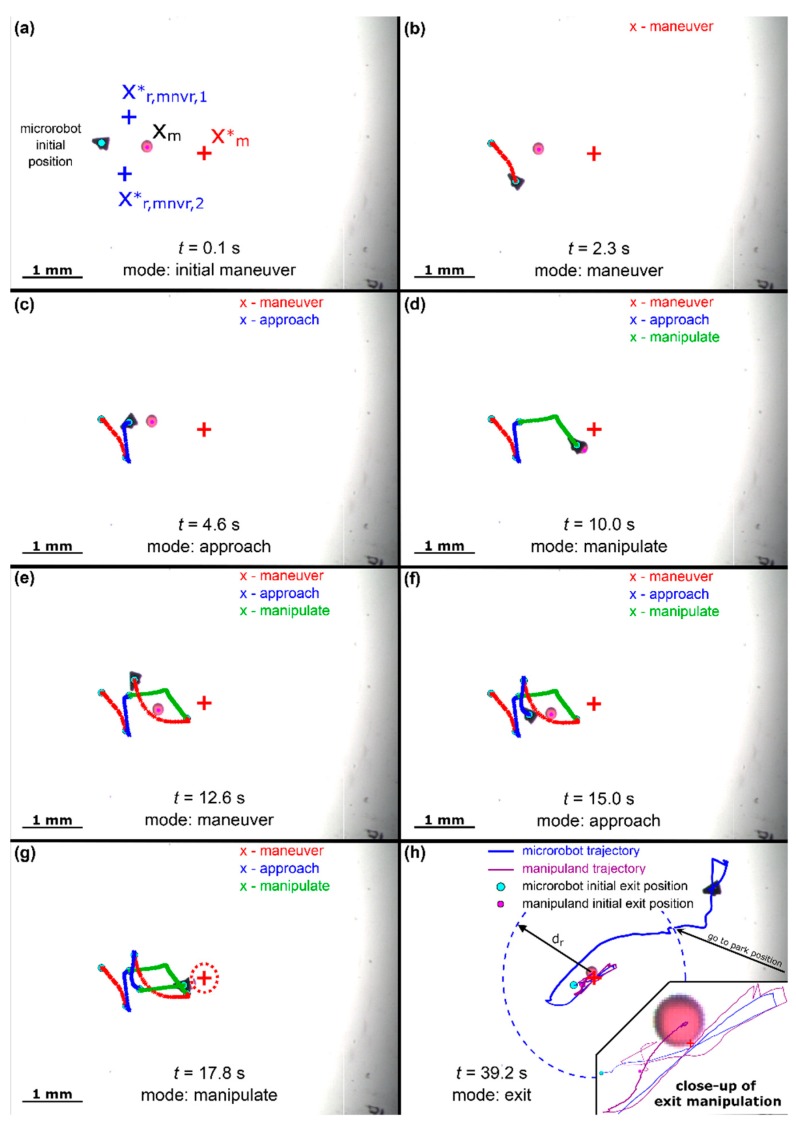
Experimental manipulation sequence to achieve exit mode status. (**a**) Initial system configuration; (**b**) microrobot transitioning to maneuver point; (**c**) microrobot moving to approach point; (**d**) microrobot in first manipulation attempt; (**e**) microrobot moving to second maneuver point; (**f**) microrobot moving to second approach point; (**g**) microrobot in second manipulation attempt; (**h**) microrobot moving to exit radius and then to the ‘parking’ point.

The pixel related parameters of Table 4 were determined based on the utilized magnification of the microscope. For example, the 200-µm sphere is approximately 25 pixels in diameter; therefore, the set point arrival tolerance was selected approximately to ½ the sphere diameter. To avoid potential collisions, $d_{∥},\;d_{⊥}$, and $d_{app}$ were selected to be approximately 1× to 2× the sphere diameter. The radius of influence parameter value $d_{r}$ (this is the distance where robot motion does not influence the manipuland) was observed in situ. Note that the negative value for $d_{m}$ in Table 4 projects the robot’s desired position along the manipuland’s error vector 5 pixels to the front of the manipuland while in manipulation mode. The lower level proportion integral values were based on commonly utilized tuning practices such as [31] and limited experimental tuning. Figure 5 displays operation of the microrobot through all modes of operation (Video demonstration aviable in Supplementary Materials).

From Figure 5, the proposed manipulation algorithm of Table 2 actuated the manipuland to within the exit mode tolerance (denoted by the green circle of radius 25 pixels Figure 3g) in 17.8 s using two cycles through the control modes. In Figure 5d, the manipulation algorithm switched from ‘manipulation’ mode back to ‘maneuver’ mode since the gradient of manipulation error was positive for more than 0.5 s (i.e., the robot was pushing the manipuland away from the desired final location). This switching is not unexpected as the robot is unable to accurately ‘pivot’ around the perimeter of the manipuland while in contact to align along manipuland error vector. Furthermore, the exit mode took approximately 21.4 s to actuate the robot to its final parking place in the northeast corner of the workspace at a location of (600,400) with a final manipuland position of (369,247) producing a normalized error of 8.3 pixels, or 66 µm. It should be noted that in this implementation of the exit strategy, the robot will proceed to the commanded park position when the robot achieves the desired distance from the manipuland regardless of the manipuland error. The algorithm could easily be modified so that the separation mode is continued while the manipuland error position (and possibly velocity) are outside a specified tolerance as was done in [5]. 

From Figure 5e, the microrobot took a parabolic/exponential shaped path to the second maneuver point. Ideally, the robot should have followed a straight line between two position points; however, the fixed proportional integral robot velocity controller may have experienced difficulties in achieving precise actuation due to the unmodeled, nonlinear interactions on the robot at the microscale. It was observed that the robot position performance was influenced differently depending on its location within the workspace. This varying disturbance may be attributed to such inconsistencies as changing viscosity of the fluid, presence of air bubbles at the fluid interface, or possibly variations in the applied magnetic field across the workspace. 

**Figure 6:**
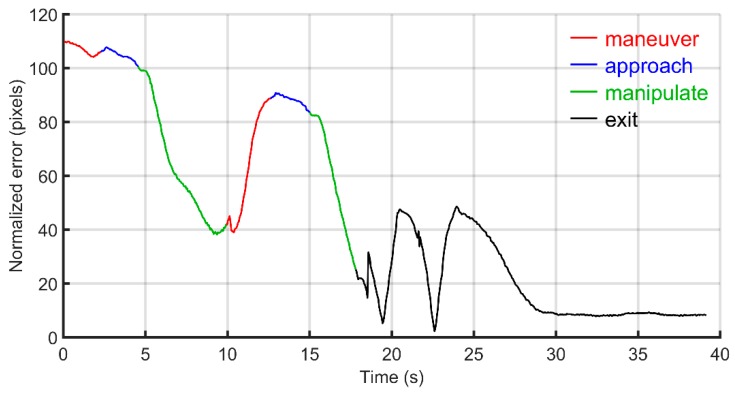
Normalization of manipuland error.

From Figure 6, one can observe that at approximately 9 s the error norm began to increase over time thereby triggering the system to reset to ‘maneuver’ mode. As the robot moved away in maneuver mode, the manipuland also followed causing significance increase in the error norm (i.e., the ‘pulling’ effect). Interestingly in ‘exit’ mode, even though the robot was trying to escape to its parking location along the manipuland error vector, the normalized error of the manipuland was further decreased from 25 to approximately 8 pixels through the interaction with the fluid (i.e., the robot was not in contact with the manipuland).

In order to demonstrate the robustness of the control method, a star shaped robot was substituted for the triangular robot, and the manipulation trial re-conducted utilizing the same control gain values of Table 4.

**Figure 7:**
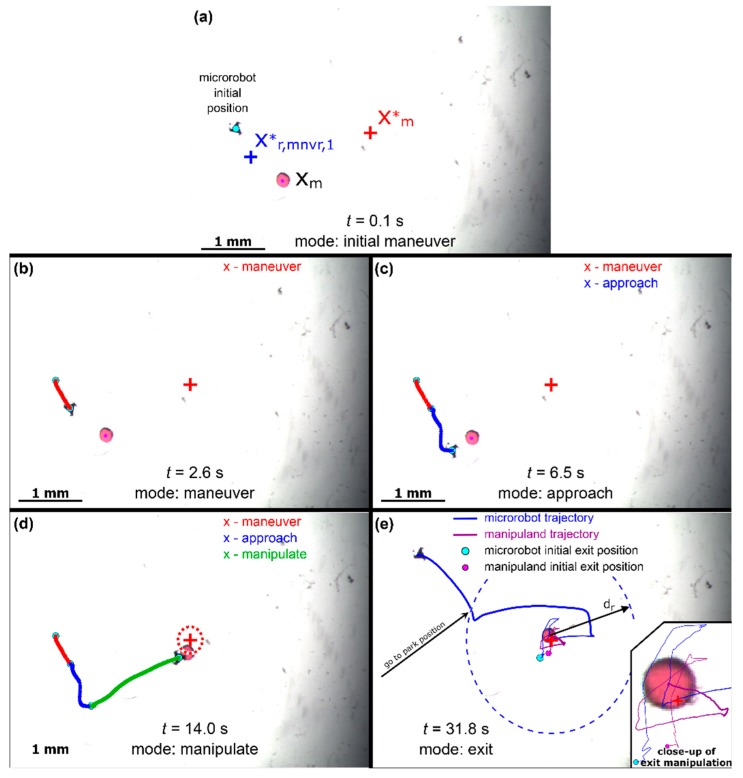
Experimental manipulation using star shaped robot. (**a**) *t* = 0.1 s, (**b**) *t* = 2.6 s, (**c**) *t* = 6.5 s, (**d**) *t* = 14.0 s, (**e**) *t* = 31.8 s.

**Figure 8:**
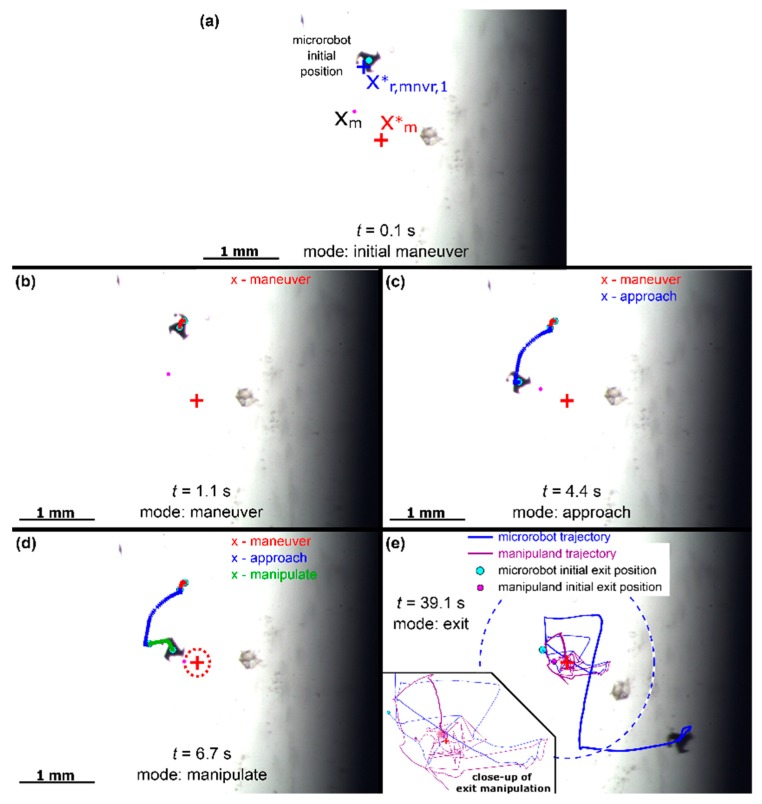
Experimental manipulation with 50-μm sphere. (**a**) *t* = 0.1 s, (**b**) *t* = 1.1 s, (**c**) *t* = 4.4 s, (**d**) *t* = 6.7 s, (**e**) *t* = 39.1 s.

From Figure 7, the proposed scheme is shown to actuate the manipuland to within 9 pixels of the desired target position while exiting the robot to its park position in 31.8 s. It should also be noted that a control cycle reset was not needed for this particular trial as the 200-µm sphere mated nicely with the microrobot protrusions. The proposed manipulation algorithm was further tested by replacing the 200-µm sphere with a smaller 50-µm bead again utilizing the gain values of Table 4 as shown in Figure 8. 

From Figure 8, the proposed method manipulated the 50-µm sphere to within 6.7 pixels of the desired target position parking the robot in 39.1 s. For this trial, algorithm stayed in exit mode of approximately 32.4 s as the robot experienced difficulty separating itself from the manipuland (this appeared be more of an adhesion phenomenon as opposed to a fluid interaction). Table 5 provides a summary of metrics over the conducted trials of the manipuland/robot control scheme.

From Table 5, the average final manipuland error over all trials is on the order of 8.33 pixels, or 66 µm. For comparison, the diameter of the manipuland sphere is approximately 25 pixels and the length of the microrobot is on the order of 30 pixels. In order to evaluate the robustness of algorithm to the low-level gain values, trials 12 and 13 of Table 5 were conducted with 75% and 125% of the values for $k_{p}$, $k_{u},\;\mathrm{and}\;k_{i}$, respectively. In trial 12, a significant increase in final manipuland error was observed when the control values are decreased by 25%. With the lower gain values, the robot was observed to experience larger position trajectory tracking errors thus not following the commanded filtered exit path. As mentioned previously, the current implementation of the exit/separation strategy does not attempt to keep the manipuland error within a select tolerance (i.e., this set of control gains may reduce error if allowed to execute longer). Future work will investigate utilizing more robust control methodologies such as that of [16] to improve positioning capabilities.

## 6. Conclusions

The experiments demonstrated that our multiphase controller can be used to direct a microrobot to position a manipuland. Average positional accuracies of 8.33 pixels (66 µm) were demonstrated over numerous trials, which is comparable to the positional accuracies described in prior work [20,22,25,26]. The performance of this controller could be improved by basing the controller mode not simply on microrobot-manipuland separation but on the measured observation of the degree to which manipuland position is affected by microrobot motion. Further improvement could also be gained by not separating the ‘approach’ and ‘maneuver’ modes, but instead utilizing a potential function approach to obstacle avoidance [32]. Further performance improvements could also be realized by combining the described multi-stage approach with an adaptive controller that would compensate for variations in the system gain parameters. The proportional integral robot velocity control system proved adequate for the described experiments in the absence of disturbances and with fixed gain values; however, the positioning performance of the robot could be further improved through utilization of more robust control methodologies such as the recursive least squares (RLS) estimation of [16]. Specifically, the adaptive nature of [16] will be better poised to compensate for the varying influences the microrobot is subject to across the entire workspace. Integration of an adaptive controller will be particularly important as we apply this control strategy to a wider variety of microrobot shapes, materials, and actuation technologies. Theoretically, this scheme should work for any two-dimensional, vision-based, microrobot-manipuland system.

## Figures and Tables

**Table 1:** Survey of prior work and comparison to present work.

Ref.	Actuation Method	Path Planning	Control Type	Release Strategy	Positioning Accuracy (%manip. size)
Floyd [19]	Contact and non-contact pushing by magnetic microrobot	Not discussed	Open loop	Not discussed	Not discussed
Zhang [20]	Non-contact pulling and pushing with rotating nanowire	Not discussed	Not discussed	Passive	~30%
Ye [21]	Non-contact entrapment by rotating 360 um spherical microrobot	Not discussed	Not discussed	Active (cease rotation)	Not discussed
El-Gazzar [22]	Non-contact pulling and pushing by a magnetic cluster	Not discussed	Teleoperation	Active (increase speed)	30–60%
Wong [23]	Capillary action at fluid interface	Not discussed	Closed loop	Not discussed	Not discussed
Munoz [24]	Laser-induced thermocapillary flow	Artificial potential fields	Closed loop	Not required	Discussed but not quantified
Rahman [25]	Microrobot bubbles actuated by laser-induced thermocapillary flow	Grasping, rotation, and translation modes	Open loop and hybrid closed loop	Active	5–10%
El-Etriby [26]	Flagellar microswimmer contact pushing	Not discussed	Teleoperation	Active (increase flagellation frequency)	55%
This work	Magnetic microrobot using contact pushing and non-contact pushing and pulling	Behavior based	Closed loop	Active (feedback driven escape vector)	33%

**Table 2:** Description of the error function for each controller mode.

Mode	Commanded robot position
Maneuver	$x_{r,mnvr}^{*}$
Approach	$x_{r,appr}^{*}$
Manipulate	$x_{r,manp}^{*}$
Exit	$x_{r,exit}^{*}$

**Table 3:** List of auxiliary variables

Symbol	Description	Equation	
$x_{m}^{*}$	Manipuland target position	Given	
$m_{∥}$	Radial unit vector	($x_{m}-x_{m}^{*})/\;|x_{m}-x_{m}^{*}|$	(1)
$m_{⊥}$	Tangential unit vector	(($x_{m}-x_{m}^{*})\cdot m_{⊥})/\;|x_{m}-x_{m}^{*}|=0$	(2)
$x_{r,mnvr}^{*}$	Maneuver targets	$x_{m}+d_{∥}m_{∥}\pm d_{⊥}m_{⊥}$	(3)
$x_{r,appr}^{*}$	Approach target	$x_{m}+d_{app}m_{∥}$	(4)
$x_{r,manp}^{*}$	Manipulate target	$x_{m}+d_{m}m_{∥}$	(5)
$x_{r,exit}^{*}$	Robot exit/separation position	$x_{m}^{*}-d_{r}m_{∥}$	(6)

**Table 4:** Experimental parameter values.

Parameter	Description	Value
$d_{tol}$	Set point arrival tolerance	10 (pixels)
$d_{∥}$	Radial offset from manipuland for maneuver point	35 (pixels)
$d_{⊥}$	Tangential offset from manipuland for maneuver point	70 (pixels)
$d_{app}$	Radial offset from manipuland for approach mode	35 (pixels)
$d_{m}$	Radial offset from manipuland for manipulate mode	−5 (pixels)
$d_{r}$	Radius from $x_{m}^{*}$ for robot to exit mode to park position	175 (pixels)
α	Filter time constant for separation/exit mode	1
$k_{p}$	Velocity gain per pixel error	2 (s^−1^)
$k_{u}$	Proportional velocity gain	10^−3^
$k_{i}$	Integral velocity gain	10^−2^

**Table 5:** Summary of experimental trials.

Trial	Initial Manipuland Error (pixels)	Initial Robot/Manipuland Separation (pixels)	Time to Exit Mode (s)	Time in Exit Mode (s)	Final Manipuland Error (pixels)	Robot/Manipuland Type
1	33.10	299.50	17.70	47.88	4.41	Wedge, 200-µm
2	28.00	275.11	8.17	72.75	3.49	Wedge, 200-µm
3	16.74	140.39	8.66	21.82	7.89	Wedge, 200-µm
4	171.38	81.63	9.34	39.50	12.26	Wedge, 200-µm
5	27.86	13.17	7.71	33.62	7.43	Wedge, 200-µm
6	5.85	100.48	16.91	9.83	9.42	Wedge, 200-µm
7	8.88	262.05	11.88	18.87	4.91	Wedge, 200-µm
8	109.71	87.85	17.86	21.29	8.31	Wedge, 200-µm
9	37.79	204.80	6.66	29.78	6.33	Wedge, 200-µm
10	102.45	134.01	8.88	14.07	8.38	Wedge, 200-µm
11	202.97	110.30	14.07	17.70	9.37	Star, 200-µm
12	150.17	226.13	25.17	40.72	26.26	Star, 200-µm
13	213.66	234.12	32.1	41.25	1.52	Star, 200-µm
14	74.77	62.67	6.72	32.38	6.70	Star, 50-µm
Avg:	84.52	159.44	13.70	31.53	8.33	

## Supplementary Materials

The following are available online at https://www.mdpi.com/2072-666X/11/2/132/s1, Video S1: usna_autonomous_microrobotic_manipulation_using_visual_servo_control.avi.
